# A Novel Nonsense Mutation in *FGB* (c.1421G>A; p.Trp474Ter) in the Beta Chain of Fibrinogen Causing Hypofibrinogenemia with Bleeding Phenotype

**DOI:** 10.3390/biomedicines8120605

**Published:** 2020-12-13

**Authors:** Tomas Simurda, Rui Vilar, Jana Zolkova, Eliska Ceznerova, Zuzana Kolkova, Dusan Loderer, Marguerite Neerman-Arbez, Alessandro Casini, Monika Brunclikova, Ingrid Skornova, Miroslava Dobrotova, Marian Grendar, Jan Stasko, Peter Kubisz

**Affiliations:** 1 National Centre of Hemostasis and Thrombosis, Department of Hematology and Transfusiology Comenius University in Bratislava, Jessenius Faculty of Medicine in Martin and University Hospital in Martin, 03601 Martin, Slovakia; jana.zolkova@gmail.com (J.Z.); simkovamonika@gmail.com (M.B.); ingrid.skornova@uniba.sk (I.S.); miroslava.dobrotova@gmail.com (M.D.); jan.stasko@uniba.sk (J.S.); peter.kubisz@uniba.sk (P.K.); 2 Department of Genetic Medicine and Development, University Medical School of Geneva, 1211 Geneva, Switzerland; rui.vilar@unige.ch (R.V.); marguerite.neerman-arbez@unige.ch (M.N.-A.); 3 Institute of Hematology and Blood Transfusion, 128 20 Prague, Czech Republic; eliska.ceznerova@uhkt.cz; 4 Biomedical Center Martin, Jessenius Faculty of Medicine in Martin, Comenius University in Bratislava, 03601 Martin, Slovakia; zuzana.snahnicanova@uniba.sk (Z.K.); dusan.loderer@uniba.sk (D.L.); marian.grendar@uniba.sk (M.G.); 5 Division of Angiology and Hemostasis, University Hospitals of Geneva and Faculty of Medicine, 1205 Geneva, Switzerland; alessandro.casini@hcuge.ch

**Keywords:** hypofibrinogenemia, Bβ-chain gene, novel nonsense mutation, protein modelling, functional analysis, bleeding phenotype

## Abstract

Congenital hypofibrinogenemia is a rare bleeding disorder characterized by a proportional decrease of functional and antigenic fibrinogen levels. Hypofibrinogenemia can be considered the phenotypic expression of heterozygous loss of function mutations occurring within one of the three fibrinogen genes (*FGA*, *FGB*, and *FGG*). Clinical manifestations are highly variable; most patients are usually asymptomatic, but may appear with mild to severe bleeding or thrombotic complications. We have sequenced all exons of the *FGA*, *FGB*, and *FGG* genes using the DNA isolated from the peripheral blood in two unrelated probands with mild hypofibrinogenemia. Coagulation screening, global hemostasis, and functional analysis tests were performed. Molecular modeling was used to predict the defect of synthesis and structural changes of the identified mutation. DNA sequencing revealed a novel heterozygous variant c.1421G>A in exon 8 of the *FGB* gene encoding a Bβ chain (p.Trp474Ter) in both patients. Clinical data from patients showed bleeding episodes. Protein modelling confirmed changes in the secondary structure of the molecule, with the loss of three β sheet arrangements. As expected by the low fibrinogen levels, turbidity analyses showed a reduced fibrin polymerisation and imaging difference in thickness fibrin fibers. We have to emphasize that our patients have a quantitative fibrinogen disorder; therefore, the reduced function is due to the reduced concentration of fibrinogen, since the Bβ chains carrying the mutation predicted to be retained inside the cell. The study of fibrinogen molecules using protein modelling may help us to understand causality and effect of novel genetic mutations.

## 1. Introduction

Fibrinogen is a complex plasma glycoprotein with a molecular weight of 340 kDa. The structure of the molecule forms a hexamer that is composed of two sets of three polypeptide chains—Aα, Bβ, and γ—joined by a disulfide bridge [1]. The mature molecule is encoded by three genes, *FGA*, *FGB*, and *FGG*, located contiguously on chromosome band 4q23. The main role of fibrinogen in hemostasis is to strengthen the platelet plug after conversion into an insoluble fibrin polymer by thrombin cleavage of fibrinopeptides A and B. The fibrin polymer traps red blood cells and platelets, leading to a stable fibrin plug that stops bleeding from the site of injury [2]. Congenital fibrinogen disorders can affect the quality and quantity of plasma fibrinogen [3]. Congenital hypofibrinogenemia is characterized by abnormally low levels of functional and antigen fibrinogen, generally due to heterozygous mutations in one of the three fibrinogen genes [4]. Most patients with hypofibrinogenemia are asymptomatic, even though according to the fibrinogen level, some patients suffer from severe bleeding and are not protected from thrombosis [5]. Mutations accounting to hypofibrinogenemia are frequently localized in the last exon 8 of *FGB*, encoding the C- terminus of the βB chain [6].

We report a novel nonsense mutation in fibrinogen Bβ chain in two unrelated patients from the Liptov region located in the north of Slovakia. They were diagnosed with congenital hypofibrinogenemia and presented a bleeding phenotype. To our knowledge, this fibrinogen variant has not been previously reported. 

## 2. Patients and Methods

### 2.1. Study Population

Our study was conducted in accordance with the Declaration of Helsinki. It was also approved by the institutional Ethics committee Comenius University in Bratislava, Jessenius Faculty of Medicine EC 26/2019, 24 April 2019, and informed consent was obtained.

### 2.2. Coagulation Tests and Rotational Thromboelastometry

Peripheral blood samples from these patients and healthy controls were collected into standard 0.125 mmol/L trisodium citrate tubes. Plate-poor plasma was then separated by centrifugation at 1500 rpm for 10 min and was used for coagulation tests. Functional fibrinogen concentration was measured by the Clauss method (IL: Instrumentation Laboratory, Bedford, MA, USA). The fibrinogen antigen was performed using turbidimetric latex immunoassay (LIA) (Hyphen BioMed, West Chester, OH, United States) from plasma samples. Prothrombin time (PT), activated partial thromboplastin time (APTT), thrombin time (TT), and reptilase time (RT) (methods and reagents from IL (Instrumentation Laboratory, Bedford, MA, United States) were measured using the Beckman–Coulter ACL TOP 550 CTS automatic analyser. Determination of the plasminogen activator inhibitor, biological activity of plasminogen, and α2-antiplasmin were measured using the chromogenic substrate method, according to the manufacturer’s instructions for the Sysmex BCS XP (Siemens Healthcare Diagnostics, Erlangen, Germany) automatic analyser. We performed a standard screening test, which measures overall fibrinolysis so-called euglobulin lysis time.

Citrated samples of blood were collected under standardized conditions, and rotational thromboelastometry (ROTEM delta, Tem Innovations GmbH, Munich, Germany) measurements were processed within a maximum of 1 h. Three routine thromboelastometry tests (EXTEM (extrinsically activated thromboelastometric test), INTEM (intrinsically activated thromboelastometric test), and FIBTEM (extrinsically activated thromboelastometric test)) were conducted to assess clotting time (CT), clot formation time (CFT), amplitude representing the clot firmness in 10 min after CT (A10), and maximum clot firmness (MCF), according to the manufacturer’s instructions.

### 2.3. Fibrin Polymerization Curve

Patient´s and control plasma samples were diluted 1:3 with TRIS buffer pH 7.4 for functional fibrinogen testing. All reactants were incubated at 37 °C. Measurements were triggered with addition of human thrombin (final concentration 0.1 NIH U/mL and 0.9 NIH U/mL). Optical density was read in duplicates after 20 s for 40 min at 350 nm by a Ceres 900 ELISA reader (Bio-tekInstruments, Winooski, VT, United States) [7].

### 2.4. Fibrinolysis

Fibrinolysis was measured in reaction mixture of plasma samples, thrombin (final concentration = 0.5 NIH U/mL), plasminogen (final concentration = 0.15 NIH U/mL), tissue plasminogen activator (final concentration 0.3 µg/mL) and CaCl_2_ (final concentration 8 mM). Patient´s and control samples were diluted 1:3 with TRIS buffer with pH 7.4. Optical density of clot formation and subsequent degradation was measured at 350 nm, using a Ceres 900 ELISA reader (Bio-tekInstruments, Winooski, VT, United States) [7].

### 2.5. Scanning Electron Microscopy (SEM)

Diluted plasma samples of patients and control (total fibrinogen 1 g/L) were incubated with thrombin (final concentration 13 U/mL) and 1 M CaCl_2_ for 3 h, then washed with cacodylate buffer and dehydrated ethanol. Subsequently, samples were dried at a critical point and coated with 10 nm layer of gold. Fibrin networks were measured in duplicates by TESCAN MIRA 3 (Tescan Brno s.r.o., Brno, Czech Republic) and evaluated with ImagineJ 1.33 software (National Institutes of Health, Bethesda, MD, United States). Fiber diameters were statistically evaluated by *t*-test.

### 2.6. Genetic Analysis and Protein Modelling

DNA was isolated from 2 mL of whole blood by a QIAamp DNA Blood Midi Kit (Qiagen, Hilden, Germany). Sequences comprising exons and exon–intron boundaries from the three fibrinogen genes (*FGA*, *FGB*, and *FGG*) were amplified by the polymerase chain reaction according to standard protocols. Dideoxy sequencing was performed using BigDye Terminator v1.1 Cycle Sequencing Kit and ABI 3500 Genetic Analyzer (Applied Biosystem, United States).

Protein modelling was performed using the Swiss-Model platform (swissmodel.expasy.org/) in automated mode [8], using the PDB file 1FZA [9] and the human fibrinogen beta chain precursor protein sequence (P02675) obtained from the UniProt database, similarly to past studies [10]. The produced models were analyzed in SwissPdbViewer 4.1.0 and POV-Ray.

## 3. Results

### 3.1. Clinical Description

Patient 1 is a six-year-old boy diagnosed with mild hypofibrinogenemia upon investigation of significant delayed bleeding after 6 h of cleft lip surgery. Treatment of bleeding consisted of administration of 1 unit of fresh frozen plasma. Except for this event, the patient did not report other spontaneous or post-traumatic bleeding episodes. Wound healing was adequate. Family history was negative for bleeding disorders.

Patient 2 is 34-year-old woman diagnosed with mild hypofibrinogenemia detected in the first trimester of her second pregnancy, complicated with a retroplacental hematoma. During the first and second trimesters, fibrinogen activity levels were 1.43 ± 0.1 g/L. Mild vaginal bleeding developed at the 24th week of pregnancy, when the fibrinogen level was 1.32 g/L. One gram (12.65 mg/kg) of fibrinogen concentrate (Haemocomplettan P, CSL Behring, Marburg, Germany) was administered, aiming at a fibrinogen level of 1.57 g/L. Pregnancy was terminated prematurely due to placental abruption with persistent contractions at the 26th week. The patient underwent a planned caesarean section by general anesthesia. Delivery was unremarkable under prophylaxis with hemostyptics (ethamsylate, aminomethylbenzoic acid). The immediate post-partum fibrinogen level was 1.57 g/L. The first pregnancy had resulted in a miscarriage in the first month, requiring a curettage complicated by mild bleeding without the need for fibrinogen supplementation. Family history was negative for a bleeding disorder.

### 3.2. Results Coagulation Test and ROTEM Analysis

**Figure 1:**
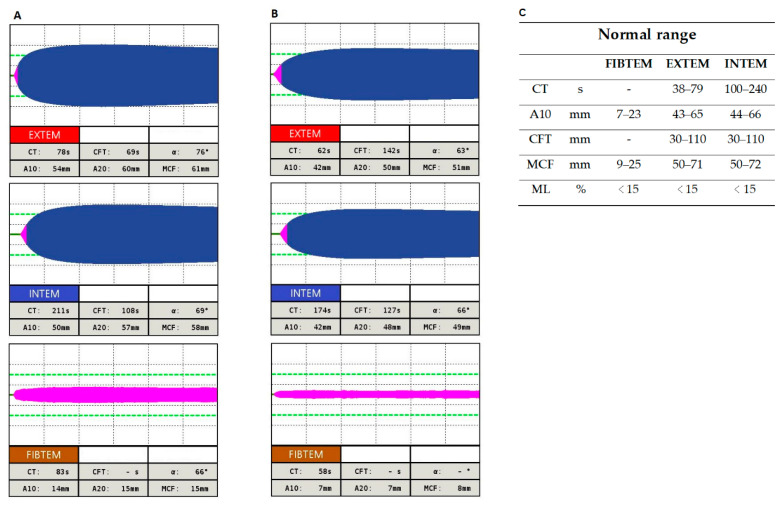
Rotational thromboelastometry EXTEM (extrinsically activated thromboelastometric test), INTEM (intrinsically activated thromboelastometric test) and FIBTEM (extrinsically activated thromboelastometric test) for patient 1 (**A**), patient 2 (**B**) and normal range: EXTEM, INTEM and FIBREM (**C**). CT: clotting time; A10: clot amplitude 10 min after CT; CFT: clot formation time; MCF: maximum clot firmness; ML: maximum lysis.

The main laboratory characteristics of basal levels (out of pregnancy, out of the perioperative period) are summarised in Table 1. A fibrinogen work-up for both patients showed mild hypofibrinogenemia. In both probands, the plasminogen activator inhibitor type I (PAI-1) level was reduced and the euglobulin lysis time (ELT) accelerated (˂8 h). ROTEM analysis in proband 1 showed a slight prolongation of the clotting time (CT) with EXTEM (78 mm; normal range: 38–72 mm) and limit value for maximum lysis (ML) with FIBTEM (14%; *N* ˂ 15%); other all parameters were in the normal range, with EXTEM, INTEM, and FIBTEM.ROTEM analyses in proband 2 showing extended CFT in EXTEM and INTEM (142 s and 127 s, respectively; *N* = 30–110 s). In INTEM and FIBTEM the MCF was slightly extended (49 mm; normal range in EXTEM: 50–72 mm; 8 mm normal range in FIBTEM: 9–25 mm). The ML value of 17% in FIBTEM showed mild hyperfibrinolysis. All other parameters are shown in Figure 1A,B.

### 3.3. Results Fibrin Clot Studies

**Figure 2:**
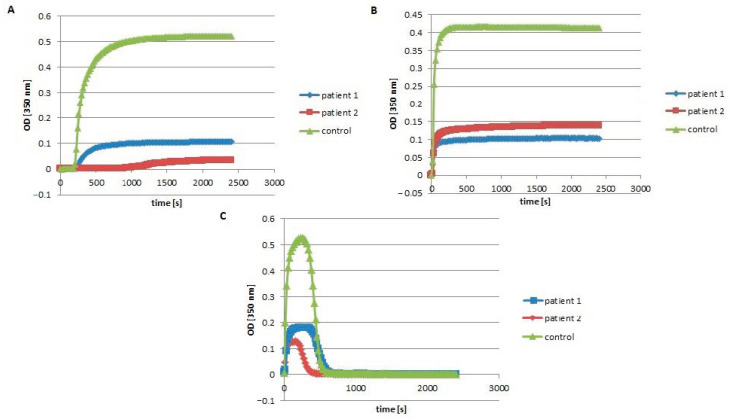
Fibrin polymerization and fibrinolysis curves. Polymerization obtained after the addition of thrombin (final concentration = 0.1 NIH U/mL): (**A**) and thrombin (final concentration = 0.9 NIH U/mL) (**B**) to the citrated plasma (patients (blue and red lines) or citrated control plasma (green line)). Fibrinolysis (**C**) in plasma patients (blue and red lines) or control plasma (green line).

As expected in the setting of hypofibrinogenemia, plasma fibrin polymerization was decreased in patients compared to controls (Figure 2A,B). In addition, patient 2 showed a markedly prolonged lag time and a shorter lysis time compared to patient 1 (Figure 2C).

**Figure 3:**
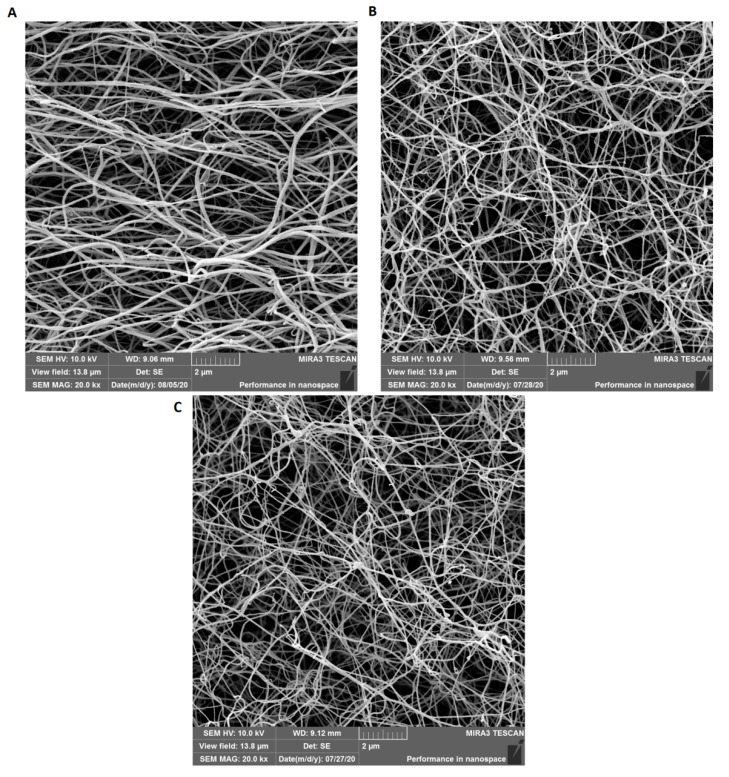
Scanning electron micrographs: (**A**) patient 1, (**B**) patient 2, and (**C**) normal control. Fibrin clots were prepared after addition of thrombin to the patients’ and control plasma.

The fibrin clot of patient 1 had denser and thicker fibers (97.6 ± 51.8 nm) compared to the control (74.9 ± 27.4 nm). The fibrin clot structure of patient 2 was very similar to the control, even though their average fiber diameter was 80.6 ± 28.8 nm (*n* = 500) (*p* = 0.05) (Figure 3A–C).

### 3.4. Results Genetic Analysis and Protein Modelling

**Figure 4:**
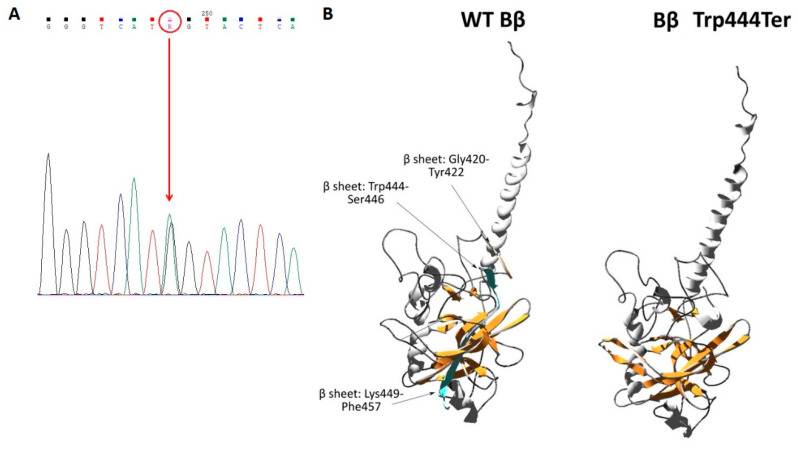
(**A**): Electropherogram of the heterozygous mutation in exon 8 of the *FGB* gene (c.1421G>A), (**B**): Analysis of the Bβ Trp444Ter mutant model. Differences between the secondary structure of the WT Bβ chain and the Bβ Trp444Ter are indicated. The amino acids missing in the mutant chain are displayed in blue. Alpha helices and beta sheets are colored in grey and orange, respectively, while the remaining residues are assigned as dark grey. Amino acids are numbered according to the mature protein sequence. Images were prepared using Swiss-PdbViewer 4.1.0, POV-Ray, and PDB file 1FZA [9].

Genetic analysis in both probands revealed a heterozygous nonsense mutation in exon 8 of the *FGB* gene c.1421G>A (Figure 4A). At the protein level, this results in the nonsense substitution of a tryptophan, with a premature termination codon at position p.474 of the Bβ chain (or 444, according to the mature chain lacking the signal peptide). This mutation was not present in the GnomAD repository (reporting data on >125,000 exomes and >15,000 genomes; https://gnomad.broadinstitute.org/). The novel mutation was named “Fibrinogen Martin IV” after the town of its discovery. The results of protein modeling showed differences between the secondary structures. These changes are marked between the wild-type (WT) Bβ chain and the Bβ Trp444Ter (p. Trp474Ter) (Figure 4B).

## 4. Discussion

We identified a new nonsense mutation in *FGB* leading to mild hypofibrinogenemia in two unrelated patients. According to the mutation database of fibrinogenic variants (http://site.geht.org/base-fibrinogene/), the majority of causative mutations for afibrinogenemia are identified in the *FGA* gene [11]. Most fibrinogen variants in hypofibrinogenemia are localized in the *FGG* gene, and only 26.6% of causative mutations are localized in the *FGB* gene [6]. There are several mechanisms that can lead to fibrinogen disorders at several levels: DNA, RNA, or protein. Various causes have been reported, such as defective assembly, decreased synthesis, secretion or increased intracellular protein degradation, or a combination of these defects [12,13]. Congenital quantitative fibrinogen disorders are most often caused by null mutations, but also missense mutations, many of which are clustered in *FGB* exon 8, encoding the highly conserved C-terminal globular domain fibrinogen Bβ [10]. Mutations in this location are known to severely impact fibrinogen assembly and secretion [14].

Clinical manifestations in hypofibrinogenemia are highly variable according to fibrinogen levels [15]. Most patients with hypofibrinogenemia are incidentally diagnosed during routine coagulation screening [1]. Most bleeding episodes are induced by trauma or surgery [16]. Pregnancy and delivery are high-risk situations in hypofibrinogenemic women, with an increased risk of miscarriages, metrorrhagia, and placenta abruption [17]. Fibrinogen replacement therapy is efficient at treating acute bleeding or preventing bleeding complications [18,19]. Laboratory analysis in our two patients confirmed mild hyperfibrinolysis by euglobulin lysis time. In addition, the ML results in FIBTEM showed borderline lysis values in patient 1 and mild hyperfibrinolysis in patient 2 (14% vs. 17%, respectively; normal range ˂ 15%). Clot lysis time was also decreased in patient 2. Fibrinolysis is a highly regulated process, starting with fibrin formation and the activation of the tissue plasminogen activator (t-PA) on plasminogen-binding sites. Release of tissue plasminogen activator (t-PA) from endothelial cells leads to the conversion of proenzyme plasminogen into plasmin [20]. Hyperfibrinolysis may develop independently of activation of the coagulation, and occurs when plasmin creation exceeds the inhibitory effect of α2-antiplasmin. The balance of fibrinolytic activators to their inhibitors is disturbed, and may result from the depletion of PAI-1 [21]. Low levels of PAI-1 were present in both patients, while plasminogen and α2-antiplasmin levels were in the physiological range, as were the D-dimers.

Hypofibrinogenemia is classified as a quantitative fibrinogen disorder [4]. In our patients, we observed decreased polymerization and abnormal clot structures, reflecting the reduced level of fibrinogen. Fibrinogen concentration is the main determinant of the structure (fiber thickness, branching, and network density) of the resulting fibrin network [22,23]. Indeed, in contrast to dysfibrinogenemia and hypodysfibrinogenemia, in hypofibrinogenemia, there is no fibrinogen variant in the circulation, so differences in clot properties are not related to the fibrinogen mutation [4]. However, other proteins or common polymorphisms, such as the FXIII Val34Leu, can influence fibrinolysis that has been associated with reduced rate of fibrinolysis [24]. On the other side, the rate of fibrinolysis is correlated with fibrinogen concentrations [25]. The enhanced fibrinolysis observed in our patients could be explained by the decreased fibrinogen levels and modifying polymorphisms.

The literature describes five heterozygous nonsense mutations related to hypofibrinogenemia in C-terminal domains of the Bβ chain (Gln339, Gln393, Trp402, Cys407, and Trp440) [26,27,28,29,30], mostly located in the exon 8 of *FGB*. In four genetic variants the clinical phenotype was severe bleeding, similar to those typically observed in patients with afibrinogenemia, such as epistaxis, umbilical cord bleeding, splenic rupture, and intracranial hemorrhage, but also milder manifestations, as described in our two patients. Patients with a nonsense mutation in the C-terminal domain of the Bβ chain had a mild hypofibrinogenemia; four patients carrying the nonsense Bβ Gln393 mutation were found to be suffering from moderate hypofibrinogenemia [27]. In addition to bleeding symptoms in a patient with the Bβ Cys407 mutation, pregnancy complications (recurrent pregnancy loss in the first trimester) were also reported [30]. Only one nonsense mutation, Bβ Trp402, was associated with an asymptomatic phenotype [29].

Protein modeling provide a better understanding of the molecular anomaly underlying the defect in fibrinogen [10]. As shown via modeling, the novel nonsense mutation is predicted to result in changes in the secondary structure of the molecule, with the loss of three β sheet arrangements (Gly420–Tyr422; Trp444–Ser446; Lys449–Phe457). Several hydrogen bonds within the molecule are also predicted to be affected, notably the ones involving the missing amino acids and surrounding residues (e.g., between Arg448 and Glu315 or Tyr416, or between Tyr445 and Trp317). These data add to previous results [14] obtained for *FGB* cDNAs encoding proteins truncated at residues very close to Trp444, i.e., Trp440Ter, a mutation also identified in heterozygosity in a patient with hypofibrinogenemia [30] and Tyr445Ter [14]. All these mutations are located in the globular C-terminus of Bβ chains. The βC domain plays a key role in the control of fibrinogen secretion [31]. In this study, using a transfected cell model system, Vu et al. showed that the deletion of more than seven residues from the Bβ C-terminus leads to inhibition of fibrinogen secretion. Assembled mutant fibrinogen was detected inside cell extracts, but not in the media, due to mutant hexamer retention. The greater extent of truncation suggests increased instability of these truncated chains [14]. Altogether, these results suggest that the secretion of Bβ Trp444Ter fibrinogen is also prevented by the endoplasmic reticulum quality control machinery concordant with the observed phenotype, i.e., mild hypofibrinogenemia when the mutation is in heterozygosity. 

## 5. Conclusions

We report a novel nonsense fibrinogen mutation in the C-terminal domains of the Bβ chain in patients with mild hypofibrinogenemia with bleeding complications. Nonsense mutations in this localization are mostly associated with moderate and mild hypofibrinogenemia. The continuous identification of novel molecular defects responsible for fibrinogen deficiency, followed by modeling of the fibrinogen mutant, helps to provide a better comprehension of the complexity of fibrinogen molecule and pathophysiological processes.

## Figures and Tables

**Table 1:** Screening and special coagulation tests of two patients with hypofibrinogenemia.

	Age	Clotting Time (s)			Fibrinogen (g/L)		ELT (h)	PAI-1 (IU/mL)	Pls (%)	α2-AP (%)
		PT	APTT	TT	Clauss	Ag				
Patient 1	6	12.2	27.5	19.8	1.43	1.40	5.0	1.10	116.6	120.0
Patient 2	34	13.0	28.3	23.8	1.06	1.10	6.0	0.50	88.4	111.8
Normal range		9.4–12.5	22–32	15–22	1.8–4.2	1.8–4.2	8.0–16.0	2.00–7.00	80.0–120.0	80.0–120.0

PT: prothrombin time; APTT: activated partial thromboplastin time; TT: thrombin time; Ag: antigen by latex immunoassay; ELT: euglobulin lysis time; PAI-1: plasminogen activator inhibitor type I; Pls: plasminogen; α2-antiplasmin: α2-AP.

## References

[1] Tiscia L.G., Margaglione M. (2018). Human Fibrinogen: Molecular and Genetic Aspects of Congenital Disorders. Int. J. Mol. Sci..

[2] Weisel J.W., Litvinov R. (2017). Fibrin Formation, Structure and Properties. Subcell. Biochem..

[3] Casini A., Undas A., Palla R., Thachil. J ., de Moerloose P. (2018). Subcommittee on Factor XIII and Fibrinogen. Diagnosis and classification of congenital fibrinogen disorders: Communication from the SSC of the ISTH. J. Thromb. Haemost..

[4] Simurda T., Zolkova J., Snahnicanova Z., Loderer D., Skornova I., Sokol J., Hudecek J., Stasko J., Lasabova Z., Kubisz P. (2018). Identification of Two Novel Fibrinogen Bβ Chain Mutations in Two Slovak Families with Quantitative Fibrinogen Disorders. Int. J. Mol. Sci..

[5] Simurda T., Casini A., Stasko J., Hudecek J., Skornova I., Vilar R., Neerman-Arbez M., Kubisz P. (2020). Perioperative Management of a Severe Congenital Hypofibrinogenemia With Thrombotic Phenotype. Thromb. Res..

[6] Casini A., Blondon M., Tintillier V., Goodyer M., Sezgin M.E., Gunes A.M., Hanss M., De Moerloose P., Neerman Arbez M. (2018). Mutational Epidemiology of Congenital Fibrinogen Disorders. Thromb. Haemost..

[7] Kotlin R., Chytilova M., Suttnar J., Salaj P., Riedel T., Santruček J., Klener P., Dyr J.E. (2007). A novel fibrinogen variant-praha I: Hypofibrinogenemia associated with gamma Gly351Ser substitution. Eur. J. Haematol..

[8] Waterhouse A., Bertoni M., Bienert S., Studer G., Tauriello G., Gumienny R., Heer F.T., de Beer T.A.P., Rempfer C., Bordoli L. (2018). SWISS-MODEL: Homology modelling of protein structures and complexes. Nucleic Acids Res..

[9] Spraggon G., Everse S.J., Doolittle R.F. (1997). Crystal structures of fragment D from human fibrinogen and its crosslinked counterpart from fibrin. Nature.

[10] Casini A., Vilar R., Beauverd Y., Aslan D., Devreese K., Mondelaers V., Alberio L., Gubert C., De Moerloose P., Neerman-Arbez M. (2017). Protein modelling to understand FGB mutations leading to congenital hypofibrinogenaemia. Haemophilia.

[11] Naz A., Biswas A., Khan T.N., Goodeve A., Ahmed N., Saqlain N., Ahmed S., Ujjan I.D., Shamsi T.S., Oldenburg J. (2017). Identification of novel mutations in congenital afibrinogenemia patients and molecular modeling of missense mutations in Pakistani population. Thromb. J..

[12] Simurda T., Snahnicanova Z., Loderer D., Zolkova J., Skornova I., Sokol J., Hudecek J., Stasko J., Lasabova Z., Kubisz P. (2016). Fibrinogen Martin: A Novel Mutation in FGB (Gln180Stop) Causing Congenital Afibrinogenemia. Semin. Thromb. Hemost..

[13] Asselta R., Duga S., Tenchini M.L. (2006). The molecular basis of quantitative fibrinogen disorders. J. Thromb. Haemost..

[14] Vu D., Di Sanza C., Caille D., De Moerloose P., Scheib H., Meda P., Neerman-Arbez M. (2005). Quality control of fibrinogen secretion in the molecular pathogenesis of congenital afibrinogenemia. Hum. Mol. Genet..

[15] Neerman-Arbez M., Casini A. (2018). Clinical Consequences and Molecular Bases of Low Fibrinogen Levels. Int. J. Mol. Sci..

[16] Peyvandi F., Haertel S., Knaub S., Mannucci P.M. (2006). Incidence of bleeding symptoms in 100 patients with inherited afibrinogenemia or hypofibrinogenemia. J. Thromb. Haemost..

[17] Cai H., Liang H., Yang J., Zhang X. (2018). Congenital hypofibrinogenemia in pregnancy: A report of 11 cases. Blood Coagul. Fibrinolysis.

[18] Simurda T., Stanciakova L., Stasko J., Dobrotova M., Kubisz P. (2015). Yes or no for secondary prophylaxis in afibrinogenemia?. Blood Coagul. Fibrinolysis.

[19] Casini A., de Moerloose P. (2020). Fibrinogen concentrates in hereditary fibrinogen disorders: Past, present and future. Haemophilia.

[20] Medved L., Tsyurupa G., Jakovlev S. (2001). Conformational changes upon conversion of fibrinogen into fibrin: The mechanisms of exposure of some cryptic sites. Ann. N. Y. Acad. Sci..

[21] Hunt B.J., Segal H. (1996). Hyperfibrinolysis. J. Clin. Pathol..

[22] Lord S.T. (2011). Molecular mechanisms affecting fibrin structure and stability. Arterioscler. Thromb. Vasc. Biol..

[23] Weisel J.W. (2005). Fibrinogen and fibrin. Adv. Protein Chem..

[24] Cushman M., Cornell A., Folsom A.R., Wang L., Tsai M.Y., Polak J., Tang Z. (2007). Associations of the beta-fibrinogen Hae III and factor XIII Val34Leu gene variants with venous thrombosis. Thromb. Res..

[25] Chapin J.C., Hajjar K.A. (2015). Fibrinolysis and the control of blood coagulation. Blood Rev..

[26] Marchi R., Brennan S., Meyer M., Rojas H., Kanzler D., De Agrela M., Ruiz-Saez A. (2013). A Novel Mutation in the FGB: C.1105C>T Turns the Codon for Amino Acid Bβ Q339 Into a Stop Codon Causing Hypofibrinogenemia. Blood Cells Mol. Dis..

[27] Castaman G., Giacomelli S.H., Duga S., Rodeghiero F. (2008). Congenital hypofibrinogenemia associated with novel heterozygous fibrinogen Bβ and γ chain mutations. Haemophilia.

[28] Hanss M., Ffrench P., Vinciguerra C., Bertrand M.A., De Mazancourt P. (2005). Four Cases of Hypofibrinogenemia Associated with Four Novel Mutations. J. Thromb. Haemost..

[29] Aung N.N., Kennedy H., Faed J.M., Brennan S.O. (2015). Novel Heterozygous Bβ (c.1311T>A) Mutation (Fibrinogen St Kilda) Associated with Recurrent Pregnancy Loss. Pathology.

[30] Homer V.M., Brennan S.O., Ockelford P., George P.M. (2002). Novel fibrinogen truncation with deletion of Bbeta chain residues 440–461 causes hypofibrinogenaemia. Thromb. Haemost..

[31] Vu D., Neerman-Arbez M. (2007). Molecular mechanisms accounting for fibrinogen deficiency: From large deletions to intracellular retention of misfolded proteins. J. Thromb. Haemost..

